# Open access EEG dataset of repeated measurements from a single subject for microstate analysis

**DOI:** 10.1038/s41597-024-03241-z

**Published:** 2024-04-13

**Authors:** Qi Liu, Shuyong Jia, Na Tu, Tianyi Zhao, Qiuyue Lyu, Yuhan Liu, Xiaojing Song, Shuyou Wang, Weibo Zhang, Feng Xiong, Hecheng Zhang, Yi Guo, Guangjun Wang

**Affiliations:** 1 Institute of Acupuncture and Moxibustion, China Academy of Chinese Medical Sciences, Beijing, China; 2 Tianjin University of Traditional Chinese Medicine, Tianjin, China; 3 Beijing Hospital of Integrated Traditional Chinese and Western Medicine, Beijing, China

**Keywords:** Preclinical research, Translational research

## Abstract

Electroencephalography (EEG) microstate analysis is a neuroimaging analytical method that has received considerable attention in recent years and is widely used for analysing EEG signals. EEG is easily influenced by internal and external factors, which can affect the repeatability and stability of EEG microstate analysis. However, there have been few reports and publicly available datasets on the repeatability of EEG microstate analysis. In the current study, a 39-year-old healthy male underwent a total of 60 simultaneous electroencephalography and electrocardiogram measurements over a period of three months. After the EEG recording was completed, magnetic resonance imaging (MRI) was also conducted. To date, this EEG dataset has the highest number of repeated measurements for one individual. The dataset can be used to assess the stability and repeatability of EEG microstates and other analytical methods, to decode resting EEG states among subjects with open eyes, and to explore the stability and repeatability of cortical spatiotemporal dynamics through source analysis with individual MRI.

## Background & Summary

Microstate analysis is an analytical approach for extracting information from electroencephalography (EEG) signals and is used to study the electrophysiology of the brain; this approach is characterized by a high temporal resolution and low costs^1^. Lehmann *et al*. demonstrated that multichannel resting-state EEG topography data remain stable for a certain period, and after the rapid transition to a different topography, they stabilize again^2,3^. These quasi-stable states are called EEG microstates and are regarded as the “atoms of brain activity”. Therefore, EEG microstate analysis is a method for extracting information from large-scale brain networks across all areas of the cortex^4^. Numerous studies have demonstrated the potential benefits of applying microstate analysis to understand brain networks and to assess health and disease. Several neuropsychiatric disorders have been detected and studied via EEG microstate analysis. Kim *et al*. validated the use of microstate features to detect schizophrenia^5^. Qin *et al*. predicted depressive symptoms in college students using EEG microstate temporal dynamics^6^. Tait *et al*. used a novel measure of complexity to observe microstate transitions and reported that EEG microstate complexity may be useful for the early diagnosis of Alzheimer’s disease^7^. EEG microstates have also been used to evaluate and explore some remedies. Liu *et al*. used microstate analysis to assess the consciousness of the brain during propofol-induced sedation^8^. Si *et al*. conducted EEG microstate analysis to obtain objective neuroimaging evidence regarding the neuromodulation effect of acupuncture combined with deqi^9^. Gold *et al*. investigated microstate changes after transcranial magnetic stimulation (TMS) and described the modulation of temporal dynamics in the antidepressant response to TMS therapy^10^. Moreover, the neural mechanisms of numerous behaviours have been explored and studied via EEG microstate analysis. Brechet *et al*. used EEG microstate analysis to examine the reorganization of brain network connectivity in several brain areas after meditation^11^. Tomescu *et al*. used correlated neuronal activity captured by microstate analysis to explain and predict social imitation-induced changes in the midst of spontaneous thoughts and behavioural states^12^. The technical robustness and experimental reliability of microstate analysis play important roles in its application and development. The repeatability of the EEG microstate method can directly influence the stability of the experimental results and thus their reliability. Therefore, an understanding of the repeatability of EEG microstate analysis is essential for its best use in both clinical and research applications. One of the methods for assessing repeatability involves the use of identical imaging equipment to image subjects multiple times in a short period^13^.

Although it has been widely applied in many studies, a shortcoming of EEG microstate analysis is that it can be influenced by many internal and external factors, such as participant preparation, ambient temperature, and equipment stability. Currently, little is known about the repeatability and stability of EEG microstate analysis, and few studies have evaluated its repeatability. Khanna *et al*. collected resting-state EEG data from 10 healthy subjects over three sessions separated by at least 48 hours to examine the reliability of the microstate features^14^. Liu *et al*. recorded two sets of 5-min resting-state EEG signals among 54 young, healthy participants on two consecutive days to investigate the reliability of the EEG microstate characteristics^15^. Popov *et al*. assessed the reliability of the main resting-state EEG metrics in a sample of 95 young individuals and 93 older individuals^16^. In addition, the recent two studies have important reference significance for the analysis of EEG microstates. One study focused on the population-level estimates of normative microstate temporal dynamics from a meta-analysis perspective and supported that microstates and their dynamics reflect functionally relevant properties of large-scale brain networks^17^. Another study evaluated the short- and long-term retest-reliability of EEG microstate characteristics in more than 500 subjects recorded 4 times each (twice within the same day and 60 days apart) and found good to excellent reliability of most microstate parameters^18,19^. These studies have examined the stability of microstates at the group level or the population level, or have explored the reliability of microstates over several measurements in the short or long term. Additionally, most of these studies were conducted by performing several repeated EEG measurements on multiple subjects; few studies performed multiple measurements on the same subject to evaluate the repeatability of EEG microstate analysis. For individuals, EEG microstates may always be in a dynamic fluctuating state, and this fluctuating state can be described in terms of both stability and variability. However, from the perspective of stability, current analyses at the group level for multiple individuals may be affected by individual differences in resting-state EEG signals with respect to age, physique and other factors; from the perspective of variability, there are no studies that have examined the fluctuating changes in one individual microstates over multiple different time scales. Therefore, both the stability of the measurements, and the variability of the microstates, require repeated measurements within a defined time frame. From this point of view, multiple repeated measurements at the individual level are important. Accordingly, we performed 60 EEG data recordings on one subject in the resting-state condition over a period of approximately 3 months.

Among the studies that examined the repeatability of EEG microstate analysis, few of them also provided the datasets used to derive their findings. This practice makes it difficult to reuse datasets and reproduce its experimental results, and these difficulties prevent greater understanding of the data from being gained. Furthermore, this practice makes it difficult to apply the data to new research. For example, if the dataset used by Liu *et al*. was freely available, it could be analysed with other methods in addition to microstate analysis to evaluate its repeatability and stability^15^. In addition, few EEG datasets with multiple repeated measurements of one individuals have been shared publicly thus far; such datasets would enable researchers to explore the repeatability of microstates and to perform EEG source localization.

In the present study, we share an organized dataset including data that were collected with the subject’s eyes open and at resting state. The data were collected from one participant at 60 time points for approximately 3 months. This dataset includes EEG, magnetic resonance imaging (MRI) and electrocardiogram (ECG) data and was used for comprehensive assessment and to validate the repeatability of EEG microstate analysis. We envision that this dataset could be used as follows. First, in addition to the repeatability assessment of the EEG microstate analysis, this dataset enables the validation of methods specific to EEG data and the evaluation of their repeatability and stability. For instance, the repeatability and reliability of EEG spectral or connectivity analysis can be assessed by applying this dataset. Second, this dataset can be applied to decode resting EEG states with eyes open using EEG-based measures as features. Third, the EEG and MRI data can be combined to perform EEG source analysis, thus enabling researchers to locate the EEG sources in anatomically defined brain structures, explore the cortical spatiotemporal dynamics of the human brain and evaluate its repeatability and stability^20^.

In the following sections, we briefly describe the participant and procedure, the EEG and MRI data acquisition methods, the data records, the technical validation, and the sharing and access policy.

## Methods

### Participant and procedure

In the present study, a 39-year-old male was recruited. He is a medical researcher with a medical education background. This subject had corrected-to-normal vision and reported being right-handed. He had no history of neurological or psychiatric diseases and participated in the study after providing written informed consent. Our experimental procedure was conducted in accordance with the Declaration of Helsinki^21^ and was approved by the Ethics Committee of the Institute of Acupuncture and Moxibustion, China Academy of Chinese Medical Sciences. The subject received monetary compensation for his participation.

**Fig. 1:**
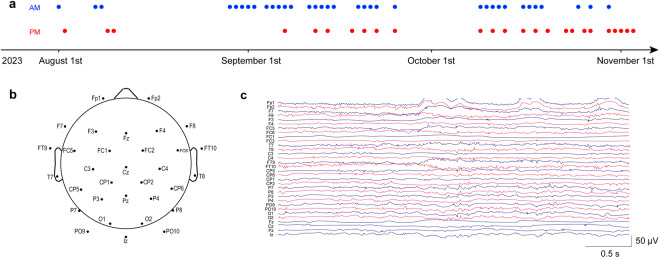
Schedule for measurements, electrode locations and EEG signal examples. (**a**) Schedule for measurements. Each blue dot represents a morning recording. Each red dot represents an afternoon recording. (**b**) Location of the 32 electrodes placed based on the International 10–20 system. (**c**) An example of a raw EEG signal displayed by channels.

The subject was advised to avoid alcohol, tea, and coffee for at least 24 hours prior to each measurement. During signal recording, the subject maintained a comfortable position in his seat. He was shown a red fixation cross at the centre of a white background placed at eye level approximately 70 cm in front of him. During the recording process, the subject was required to stare at the red fixation cross with his eyes open for 6 minutes. He then completed numerous tasks and multiple simultaneous signals were recorded (the results of these tasks and recordings will be reported elsewhere). This subject was asked to complete 60 recordings, and the schedule for signal recording is shown in Fig. 1a.

### EEG data acquisition

The EEG data were recorded with a 32-electrode EEG recording cap (Easycap GmbH, Germany) and a 24-bit EEG amplifier (NeurOne EXG, Bittium, Finland) in DC mode at a sampling rate of 20 kHz^22^. The recording electrodes were located at standard locations following the International 10–20 system^23^. FCz and AFz served as the reference and ground electrodes, respectively^24^. Figure 1b shows the placement of the 32 active electrodes used for the experiment. The EEG signal was digitized using NeurOne software, and Fig. 1c shows an example of a raw EEG image. The scalp of the subject was prepared for EEG recording using a conductive gel (Quick-Gel, Neuromedical Supplies®)^25^. The electrode impedance was generally reduced to below 5 kΩ prior to data collection. The subject was instructed to relax and avoid any specific mental operations.

Moreover, the NeurOne system was simultaneously used to record a pair of vertical electrooculograms (VEOGs) and a pair of horizontal electrooculograms (HEOGs) at supra/suborbital and external canthi sites, respectively, which may be useful for removing ocular artefacts during a later processing step^26^. Finally, two cutaneous electrodes were connected to the same system to acquire the ECG data.

### MRI data acquisition

To construct accurate forward models for source analysis, the subject underwent an anatomical T1-weighted magnetization-prepared rapid gradient MRI scan (echo time/repetition time = 3.9 ms/600 ms, slice thickness = 1 mm with no gap, field of view = 250 and matrix size = 256 × 256) after all EEG recording was completed. The MRI procedures were performed using a SIEMENS Amira 1.5-T scanner with a 16-channel phased array head coil. Three-dimensional sampling perfection with application-optimized contrasts using different flip angle evolutions (3D-SPACE) scans was also performed.

## Data Records

**Fig. 2:**
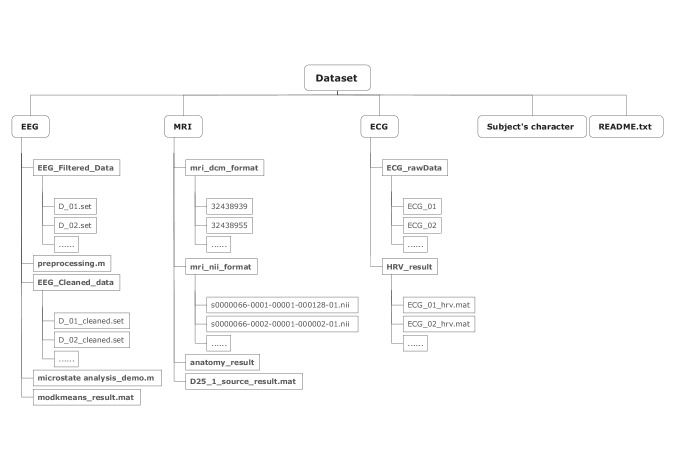
Final dataset structure, files, and naming.

All the filtered and processed EEG, MRI and ECG data used in this work are available at Figshare^27^ (10.6084/m9.figshare.24877770.v3). The current dataset contains 3 folders, namely, “EEG”, “MRI”, and “ECG”, and two files, namely, “Subject’s character.xlsx” and “README.txt”. A description of the different folders is provided in the following paragraph, and Fig. 2 shows the dataset description.

The “EEG” folder contains two subfolders, named “EEG_Filtered_Data” and “EEG_Cleaned_data”, which include filtered EEG data and cleaned EEG data, respectively. The EEG data for each measurement is saved in the.set and.fdt file formats (two files per measurement). In addition, the “EEG” folder contains EEG preprocessing (preprocessing.m) and microstate analysis (microstate analysis_demo.m) scripts. The microstate analysis results are also provided as “modkmeans_result.mat”. In the “MRI” folder, there are three subfolders. In each subfolder, there are two folders, namely, “mri_dcm_format” and “mri_nii_format”, which contain MRI data in.nii format and.dicom formats, respectively. The header model data processed by the Cat12 and Brainstorm software packages are saved in the “anatomy_result” folder. Additionally, the source results of the EEG data from the 25th EEG measurement are saved as “D25_1_source_result.mat” in the “MRI” folder. In the “ECG” folder, two subfolders, namely, “ECG_rawData” and “HRV_result”, include the raw ECG data from 60 measurements and the results of heart rate variability analysis, respectively. In addition to these 3 folders, the subject’s information is included in the “Subject’s character.xlsx” file.

## Technical Validation

### EEG data preprocessing

The EEG data processing toolbox EEGLAB^28^, which was developed by Delorme & Makeig, was used. The original EEG data were exported in European data format (EDF) and resampled at 500 Hz^29^. The data were then high-pass filtered at 0.5 Hz and low-pass filtered at 70 Hz. After this step was completed, the EEG data files were saved to the “EEG_Filtered_Data” folder in the.set and.fdt formats^27^.

Then, the cleanLineNoise.m function was used to denoise the data, average the reference, and perform bandpass filtering (2–30 Hz). Subsequently, the EEG time series were segmented into 1-s epochs, after which the bad epochs were removed. Independent component analysis (ICA) was subsequently applied to eliminate artefacts caused by eye blinks, eye movements, or jaw clenching^30^. ICA components were manually inspected, and the components corresponding to the noise and artefacts were excluded. After this step was completed, the cleaned EEG data files were saved to the “EEG_Cleaned_data” folder in the.set and.fdt formats^27^.

### Microstate analysis

**Fig. 3:**
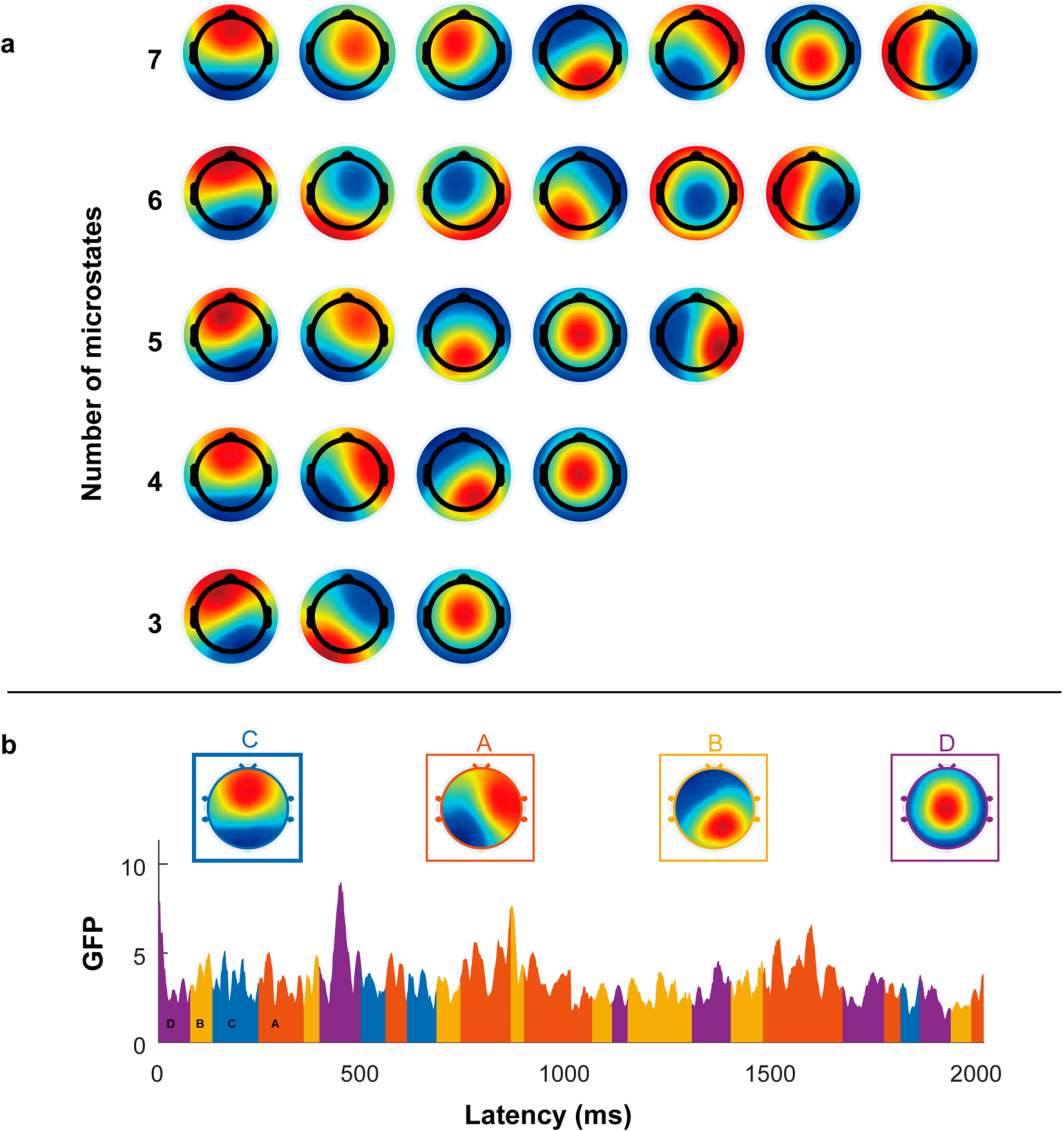
Resting-state EEG microstates. (**a**) Prototype topographies from 3 to 7 clusters. (**b**) The dynamic process of microstate changes over time. Elucidation of the GFP signal of active microstates for 2000 ms, from 0 ms to 2000 ms.

We performed microstate analysis of preprocessed EEG data using the Microstate EEGLAB Toolbox^31^. The analysis flowchart is illustrated in Fig. 3. Initially, the global field power (GFP) was extracted from the EEG signals. Considering that the local maximum (peak) of GFP exhibits a favourable signal-to-noise ratio and stable changes in EEG topography, we set a minimum interval of 10 ms between consecutive GFP peaks and extracted brain topographic maps (also known as original maps) at 2000 randomly selected GFP peak locations. GFP peaks that exceeded 1 times the standard deviation were removed because such peaks often contain artefacts of nonneuronal origin. Subsequently, all the obtained original maps were input into a modified k-means clustering algorithm (ignoring polarity). We employed an optimized segmentation scheme with 1,000 iterations to obtain the best clustering. The number of prototype microstates can be determined based on a cross-validation criterion^32^. Figure 3a displays 3 to 7 prototype microstates for the current dataset. We identified 4 prototype microstates for resting-state EEG signals since 4 topographies have been reported to be the most common and highly reproducible for resting-state EEG signals^3,5,33,34^. The four optimal prototype microstates are Class A, Class B, Class C, and Class D. Next, four prototype microstates underwent back fitting to all the EEG recordings. Each EEG time point was categorized as one of the four prototype microstates, thereby transforming the EEG signals into a microstate sequence. The back fitting process is shown in Fig. 3b. Finally, the microstate sequence underwent temporal smoothing, and microstates with durations shorter than 30 ms were excluded to enhance the fitting quality^31^.

**Fig. 4:**
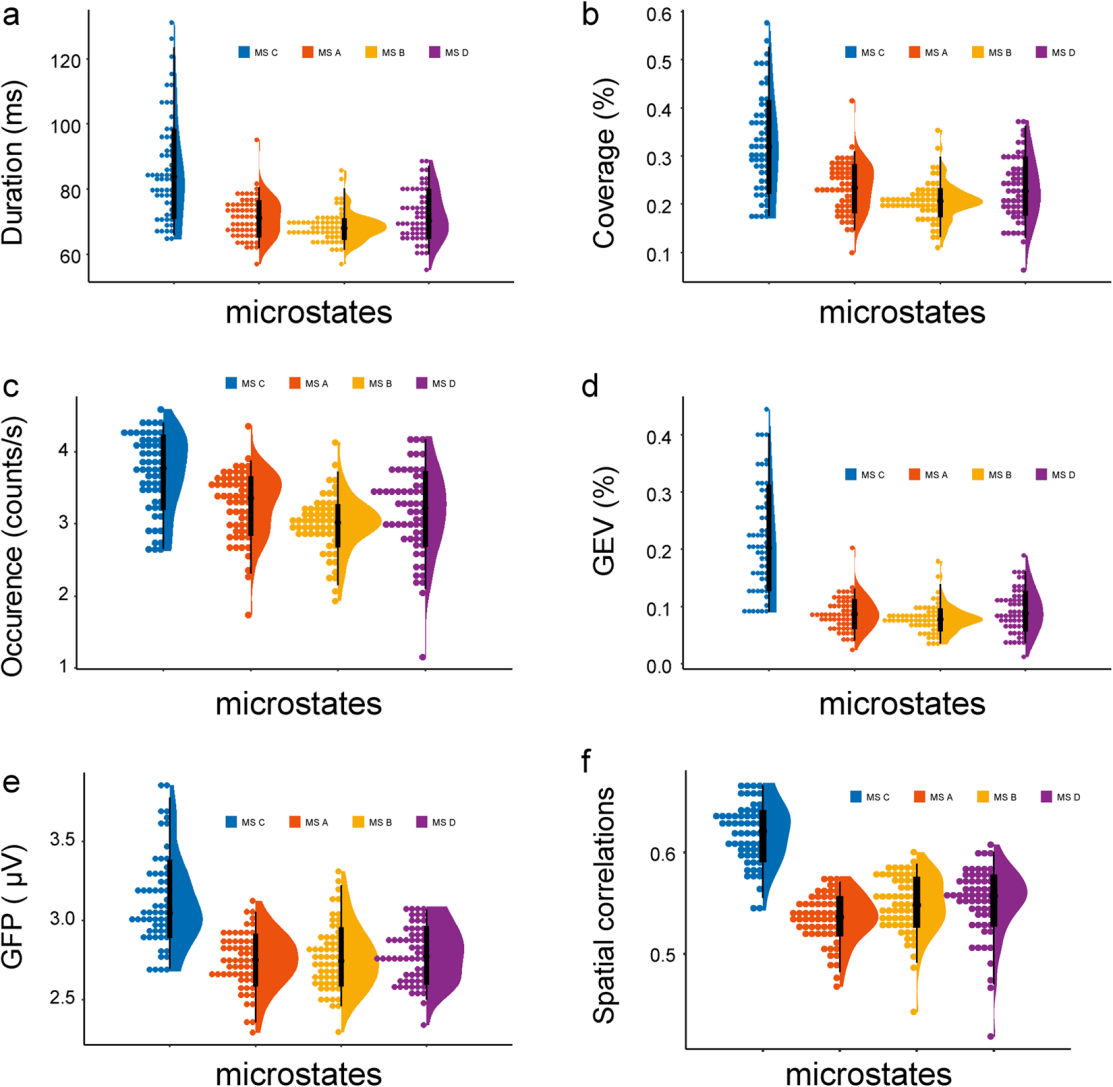
Descriptive statistics of the microstate characteristics of 60 measurements for each prototype microstate. (**a**) Mean duration. (**b**) Time coverage. (**c**) Occurrence rate per second. (**d**) GEV. (**e**) GFP. (**f**) spatial correlations. The different colours represent the different prototype microstates. Histograms show the distribution of the measured values of a continuous variable^45^. Box plots depict the sample median as well as the first (Q1) and third (Q3) quartiles^45^. The probability density functions (PDFs) indicate the distribution of a continuous probability distribution that falls among a set of values^46^.

Following the completion of the microstate analysis, five characters were computed for each of the four prototype microstates in every measurement; Fig. 4 shows the descriptive statistics of the 5 characteristics of the 60 measurements for each prototype microstate^2,35^. Figure 4a shows the distribution of the mean duration of 60 measurements for each prototype microstate; the mean duration corresponds to the uninterrupted average duration of each microstate. Figure 4b displays the distribution of the coverage of 60 measurements for each prototype microstate; the coverage represents the proportion of time that each microstate occupies throughout the recording. Figure 4c illustrates the distribution of occurrence of 60 measurements for each prototype microstate; the occurrence represents the frequency at which each microstate occurs within a one-second interval. Figure 4d shows the distribution of the global explained variance (GEV) of 60 measurements for each prototype microstate, which is quantified as the percentage of similarity between the EEG and the assigned microstates. Figure 4e represents the distribution of GFP of 60 measurements for each prototype microstate, which represents the instant strength of the electric field over the brain. Figure 4f represents the distribution of the average spatial correlations (between microstate prototype maps and their assigned EEG samples) of 60 measurements for each prototype microstate. The calculation process of spatial correlations is embedded in the toolbox and detailed in the explanation provided by Andreas Trier Poulsen^31^. The calculation formula is as follows.

$$Cor{r}_{k}=\frac{1}{{N}_{k}}\mathop{\sum }\limits_{n}^{N}Corr({a}_{k},{x}_{n})=\frac{1}{{N}_{k}}\frac{\left|{x}_{n}\cdot {a}_{k}\right|}{\left\Vert {x}_{n}\right\Vert \cdot \left\Vert {a}_{k}\right\Vert }$$

Where *Corr*_*k*_ is the spatial correlation. *N*_*k*_ is the number of samples assigned to cluster *k*. *a*_*k*_ is the prototypical map for *k’*th microstate cluster. *x*_*n*_ is the *n’*th time sample of the recorded EEG where was assigned to the cluster *k*.

### EEG source analysis

Prior to the data analyses, MRI images were converted to the.nii format from the.dicom format using the SPM12 software package^36^ (https://www.fil.ion.ucl.ac.uk/spm/software/spm12/). The Computational Anatomy Toolbox^37^ (https://www.nitrc.org/projects/cat/) was used to segment the entire brain from the raw MR image.

**Fig. 5:**
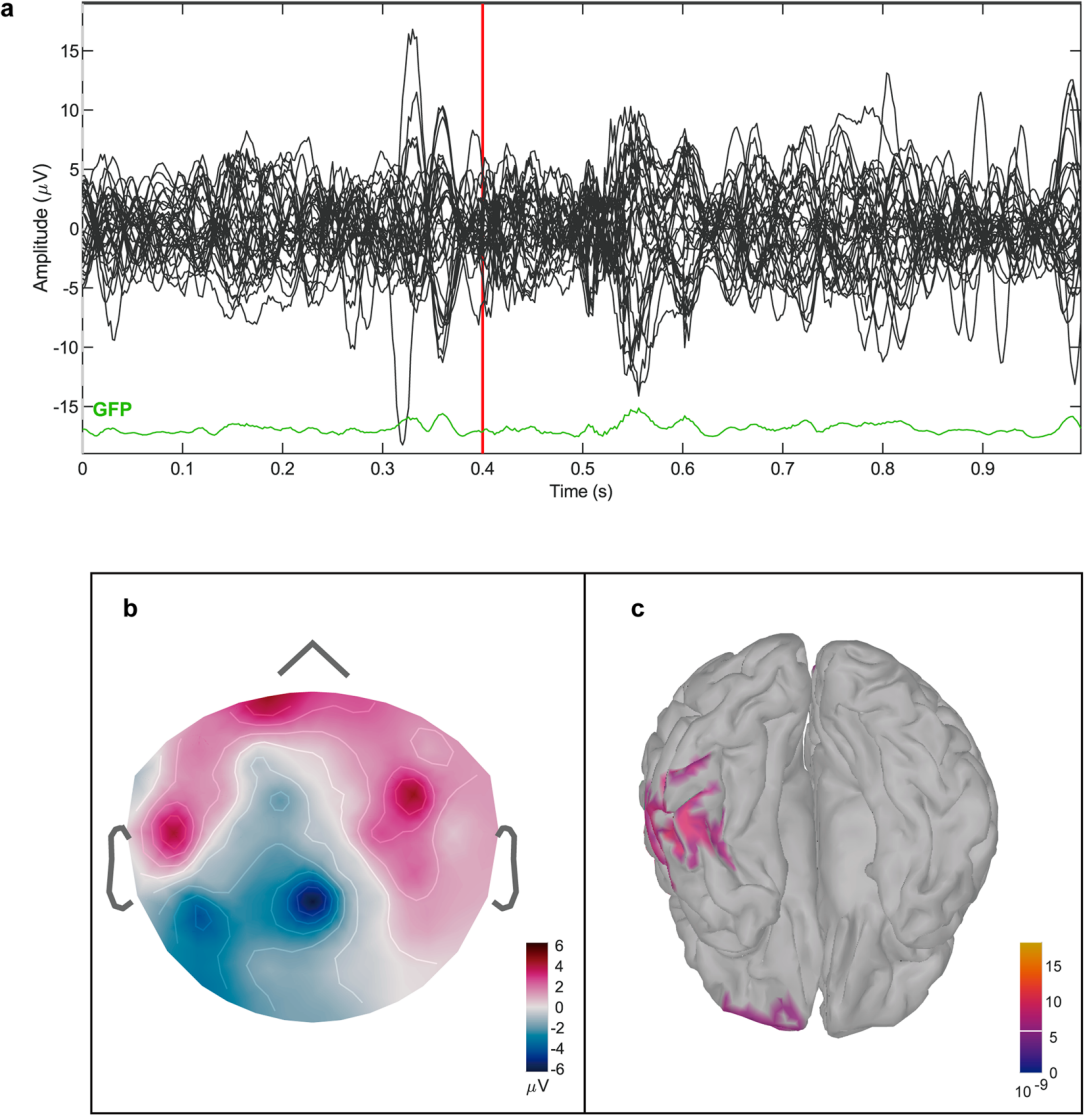
Source analysis results. (**a**) Butterfly diagram and GFP signal in the EEG data. (**b**) EEG topography at 0.4 s. (**c**) Cortical activity at 0.4 s.

The source localization of the EEG signals was completed with the Brainstorm package (http://neuroimage.usc.edu/brainstorm), and the process was carried out according to previous methods^38^. We created a new study or protocol in the Brainstorm database, and we added a new subject. Then, we imported all the MRI data preprocessed by CAT12, including individual structural MR images, cortical surface data and anatomical atlas data registered to the individual anatomy. The scalp surface was automatically reconstructed via Brainstorming from the T1 volume data. Then, the cleaned EEG files were added to Brainstorm. The registration of the EEG sensors with the structural MRI data was initialized. A forward model was then calculated for each subject using the boundary element method, as implemented in OpenMEEG^39,40^, which works within Brainstorming. Then, we conducted source estimation on the first epoch of data from the 25^th^ measurement. The source location result was named “D25_1_source_result.mat” and saved in the “MRI” folder^27^. The EEG topographic map at 0.4 seconds is shown in Fig. 5b, and the source is shown in Fig. 5c. In addition, Fig. 5a shows the butterfly diagram and GFP signal in the EEG data.

### HRV analysis

The analysis method can be found in a previous study^41^. The raw data were exported in EDF format, imported into Kubio HRV software (Kubios Oy, Kuopio, Finland) and analysed^42–44^. The data were detrended using the smoothness prior approach with a lambda value of 500, and artefacts were corrected by applying the medium filter provided by Kubios HRV.

## Data Availability

No custom code was used to generate or process the data described in the manuscript.
